# Plasma Pentosidine and Its Association with Mortality in Patients with Chronic Kidney Disease

**DOI:** 10.1371/journal.pone.0163826

**Published:** 2016-10-04

**Authors:** Anna Machowska, Jia Sun, Abdul Rashid Qureshi, Naohito Isoyama, Paul Leurs, Björn Anderstam, Olof Heimburger, Peter Barany, Peter Stenvinkel, Bengt Lindholm

**Affiliations:** 1 Renal Medicine and Baxter Novum, Karolinska Institutet, Stockholm, Sweden; 2 Department of Urology, Yamaguchi University, Ube, Yamaguchi, Japan; 3 Admiraal de Ruyter Hospital, Goes, The Netherlands; The University of Tokyo, JAPAN

## Abstract

**Background:**

Circulating advanced glycated end-products (AGEs) including pentosidine accumulating in chronic kidney disease (CKD) patients due to retention and increased formation are thought to contribute to cardiovascular disease (CVD). Here we evaluated factors linked to increased plasma pentosidine and its association with mortality in patients with different stages of CKD and undergoing different treatments.

**Methods:**

Plasma pentosidine, biomarkers of inflammation, oxidative stress and nutritional status were investigated in CKD 1–2 (n = 37), CKD 3–4 (n = 54), CKD 5 non-dialyzed (CKD5-ND; n = 386), peritoneal dialysis (PD; n = 74) and hemodialysis (HD; n = 195) patients. Factors predicting plasma pentosidine were analysed by multivariate regression analysis and mortality risk was assessed by GENMOD procedure.

**Results:**

Plasma pentosidine levels, which were higher in CKD5-ND, PD and HD groups than in CKD 1–2 group, were significantly lower in PD than in HD patients, and not different between PD patients and CKD5-ND patients. Pentosidine associated inversely with glomerular filtration rate (GFR), and additionally in PD with 8-hydroxy-2‘-deoxyguanosine (8-OHdG), and interleukin 6 (IL-6); in HD with age, IL-6 and body mass index (BMI); in CKD5-ND with age, 8-OHdG, IL-6, high-sensitive C-reactive protein (hsCRP), and soluble vascular cell adhesion protein-1 (sVCAM-1); in CKD 3–4 with 8-OHdG and sVCAM-1; and in CKD 1–2 with age and sVCAM-1. In multivariate analysis, age (one standard deviation, 1-SD higher), malnutrition (subjective global assessment, SGA), oxidative stress (8-OHdG, 1-SD higher), and belonging to CKD5-ND, HD and PD cohorts associated with 1-SD higher pentosidine. In GENMOD, 1-SD higher pentosidine independently predicted all-cause mortality (relative risk, RR = 1.04; 95% confidence interval, CI, 1.01–1.08, p = 0.01) and CVD mortality (RR = 1.03; 95% CI, 1.01–1.06, p = 0.03) after adjusting for all confounders.

**Conclusions:**

Plasma pentosidine is markedly elevated in CKD and associates with low GFR, oxidative stress and inflammation, and is an independent predictor of mortality in CKD patients.

## Introduction

In patients with chronic kidney disease (CKD), the decline of renal function associates with increasing mortality, mainly due to cardiovascular disease (CVD), especially when glomerular filtration rate (GFR) falls below 60 ml/min. Traditional cardiovascular risk factors such as advanced age, hypertension and diabetes are common, and the prognosis is further worsened—especially in end-stage renal disease (ESRD) patients—by cardiovascular complications linked to novel and uraemia-related risk factors such as inflammation, endothelial dysfunction, oxidative stress, fluid overload and vascular calcification [1, 2].

Advanced glycated end-products (AGEs) such as pentosidine, N-carboxymethyl-lysine (CML), and N-carboxyethyl-lysine are produced by covalent binding of amino groups with glucose or other saccharide derivatives during the non-enzymatic Maillard reaction and may contribute to CVD and other long term complications in CKD. AGEs accumulate in CKD patients as a consequence of enhanced formation of AGEs, due to hyperglycaemia, oxidative stress, and inflammation, and reduced disposal of AGEs by the kidneys [3–5].

A high circulating pentosidine level has been reported to associate with inflammation, malnutrition, CVD and poor clinical outcomes [6–13] but the contribution of pentosidine to the development of cardiovascular events and mortality in CKD patients has been disputed [14] and traditional risk factors in ESRD patients have been reported to be more important for cardiovascular outcomes than elevated levels of AGEs [15].

In this study, we measured plasma pentosidine in 746 patients with different stages of CKD and undergoing different dialysis treatments including peritoneal dialysis (PD) and hemodialysis (HD), explored factors potentially linked to an increased level of pentosidine, and analysed the association of pentosidine with cardiovascular and all-cause mortality.

## Materials and Methods

### Patients and study design

This study is based on post hoc analysis of baseline data and subsequent longitudinal follow-up of 746 patients with different CKD stages and undergoing PD or HD treatment. There were 37 individuals with CKD stage 1–2, 54 CKD stage 3–4 patients, 386 CKD stage 5 patients who were investigated prior to starting on dialysis (CKD5-ND), 74 prevalent PD patients, and 195 prevalent HD patients. The Ethics Committee of Karolinska Institutet, Stockholm, Sweden approved the original studies from which this study used data/samples and the study was conducted in adherence to the Declaration of Helsinki after informed written consent was obtained from each individual.

In the following, included cohorts are described briefly.

#### CKD stage 1–2

This cohort comprised 37 individuals from a population based sample randomly selected by Statistics Sweden (a government agency) from the Stockholm region, and recruited from February 2003 until April 2004, and who were found to have signs of mild CKD (macro- or microalbuminuria or reduced GFR). This cohort was created to provide an appropriate control group for the CKD stage 3–4 patients and thus they have similar age and gender distribution as the CKD stage 3–4 patients, see below. Their median age was 68 years, 70% were males and prevalence of diabetes and CVD was 0% and 8% respectively, while 6% of participants were classified as malnourished (subjective global assessment, SGA>1). Their median (10th to 90th percentile) eGFR was 79 (68–111) ml/min/1.73^2^. This group representing a population based sample and thus in most cases consisting of subjects with only mild decrease of renal function served as reference group.

#### CKD stage 3–4

This cohort consists of 54 CKD stage 3–4 patients recruited from December 2001 until March 2004. The description of the study was presented previously [16]. The median age was 60 years, 74% were males, 21% of patients were malnourished, 44% had diabetes and 26% had CVD. The most common causes of CKD were diabetic nephropathy (25%), glomerulonephritis (23%) and others (39%). Their median eGFR was 27 (15–43) ml/min/1.73^2^. Calcium channel blockers were used by 41% of patients, β-blockers by 49%, angiotensin-converting enzyme inhibitors (ACEI) or angiotensin II receptor blockers (ARB) by 50%, statin treatment by 30% and 54% of the patients received iron supplementation.

#### CKD5-ND

In total 386 patients, recruited in conjunction with initiation of dialysis therapy, were included in the study, and recruited from June 1994 until October 2012. The description of the study was presented previously elsewhere [17, 18]. The median age of patients was 55 years, 60% were males, 28% were diabetics, 31% had CVD and 30% were malnourished (SGA>1), and median eGFR was 6 (4–10) ml/min/1.73^2^. Calcium channel blockers were used by 45% of patients, β-blockers by 59%, ACEI or ARB by 54% of patients, 21% of patients received statins and 65% iron supplementation. The most common causes of CKD were diabetic nephropathy (25%), glomerular nephritis (23%) and other (39%).

#### Prevalent PD

A total of 74 prevalent PD patients were recruited from March 2008 to April 2012 in the MIMICK2 (Mapping of Inflammation Markers in Chronic Kidney Disease 2). The description of the study was presented previously [19]. The median age of patients was 61 years, 64% were males, 20% had diabetes, 22% had CVD and 42% were malnourished (SGA score >1), and median eGFR was 6 (4–9) ml/min/1.73^2^. Data on transport status were available for 40 patients; 8 were slow/average, 22 were high-average and 10 were fast/high transporters. Continuous ambulatory peritoneal dialysis (CAPD) was used by 75% of patients and 25% of the patients were treated by automated peritoneal dialysis (APD). Causes of CKD included glomerulonephritis (13%), diabetic nephropathy (12%), and hypertension/renal vascular disease (12%). Most patients were on anti-hypertensive medication with β-blockers (68%), calcium channel blockers (31%), ACEI or ARB (69%), and most received phosphate and potassium binders, diuretics, and supplementation with vitamin B, C, and D, while 35% of the patients were taking statins and 29% iron supplementation.

#### Prevalent HD

In total 195 prevalent HD patients were included in the study, and were recruited from September 2003 until September 2004 in the MIMICK1 study (Mapping of Inflammation Markers in Chronic Kidney Disease 1). The description of the study was presented previously [20]. The median age was 64 years, 57% were males, 23% had diabetes, 61% had CVD, and 45% were malnourished (SGA>1). GFR was not assessed; however, most of the patients in this cohort were functionally anuric with no or minimal residual renal function (RRF). The most common causes of CKD were glomerulonephritis (20%), diabetic nephropathy (18%) and hypertension/renal vascular disease cause (13%) and other (49%). Most patients were on anti-hypertensive medications such as β-blockers (48%), calcium channel blockers (27%), ACEI or ARB (34%) and 32% were taking statins. Most of the dialysis patients received medications such as phosphate and potassium binders, diuretics, and vitamin B, C, and D supplementation and 67% received iron supplementation.

### Blood sampling and laboratory analysis

After an overnight fast (except for HD patients), plasma samples were taken and stored at -70°C, if not analysed immediately. Plasma pentosidine was analysed by reverse-phase high performance liquid chromatography (HPLC) as described previously [6]. Although studies using specific ELISA for plasma pentosidine have shown results comparable with those obtained with HPLC assay, the latter is a more accurate method for measuring the total pentosidine content because ELISA methods may not recognize pentosidine on the interior of proteins. Furthermore, ELISA methods require chemical hydrolysis and enzymatic digestion of protein, which may alter the epitope recognized by the antibodies [6]. Circulating pentosidine is mainly present in protein bound form with albumin [7]. Therefore, in the present manuscript, the total (free plus protein bound) plasma pentosidine concentration measured in nmol/L was corrected for albumin and expressed as nmol of plasma pentosidine per gram of albumin, and this value was used for all statistical analyses.

Circulating levels of albumin (bromcresol purple), cholesterol, creatinine and high-sensitivity C-reactive protein (hsCRP, nephelometry) were analyzed using certified methods in the Department of Clinical Chemistry, Karolinska University Hospital Huddinge. Concentration of interleukin-6 (IL-6) was measured on an Immulite immunoassay Analyzer (Siemens Healthcare, Erlanger, Germany), using assays manufactured for this analyzer and according to the manufacturer’s instructions. The soluble form of vascular cell adhesion molecule 1 (sVCAM-1), a marker of endothelial activation, was analyzed by commercial ELISA kits (R&D Systems Europe, Ltd, United Kingdom). 8-hydroxy-2′-deoxyguanosine (8-OHdG) was measured using a commercial competitive enzyme-linked immunosorbent assay kit (Japan Institute for the Control of Aging, Shizuoka, Japan), following the manufacturer’s instructions.

GFR was assessed in all patients; in CKD stage 5 (n = 309) and PD cohorts (n = 49) by the mean of renal urea and creatinine clearances from a 24-hour urine collection; in CKD stage 1–2 and CKD stage 3–4 cohorts GFR by iohexol clearance; and in all patients—for comparative reasons–also by the Chronic Kidney Disease Epidemiology Collaboration (CKD-EPI) formula [21]

#### Nutritional status

Body mass index (BMI) was calculated as the body weight in kilograms divided by the square of patient height in meters. SGA was used to evaluate the overall protein-energy wasting (PEW) as described previously [22]. Briefly, SGA included six subjective assessments, three were based on the patient’s history of weight loss, incidence of anorexia or incidence of vomiting, and three were based on subjective grading of muscle wasting, presence of oedema and loss of subcutaneous fat. Then, each patient was given a score reflecting the overall nutritional status: 1 = normal nutrition, 2 = mild PEW, 3 = moderate PEW and 4 = severe PEW. In the current study, PEW was defined as SGA score >1.

### Statistical analyses

Data are expressed as median (10th to 90th percentile) or percentage, or as relative risk (risk ratio), RR, and 95% confidence intervals (95% CI) as appropriate. Statistical significance was set at the level of p<0.05. For comparisons between three or more groups we used Kruskal-Wallis ANOVA test, followed by Dunn’s test. Chi-square test was used for nominal variables. Non-parametric Spearman rank correlation analysis was used for continuous variables. To study the associations of 1 standard deviation (1-SD) higher pentosidine with other parameters we used linear multivariable regression analysis. To ascertain the adjusted RR for death associated with 1-SD higher pentosidine, multivariable GENMOD regression analysis was performed. Age, gender, diabetes (DM), SGA, hsCRP, 8-OHdG and patient cohorts were included in the model. A multiple imputation of missing values was performed using the function PROC MI, with all variables in the covariate section used to produce the values for imputation. The results for each imputation were generated by using PROC MIANALYZE and GENMOD regression analysis. Receiver operating characteristics (ROC) curves were analysed allowing calculation of areas under the curves (AUCs) and cut-off values for pentosidine in relation to all-cause and CVD-related deaths. Statistical analyses were performed using statistical software SAS version 9.4 (SAS Campus Drive, Cary, NC, USA).

## Results

### Baseline characteristics and pentosidine levels

The studied population consisted of 746 patients with different stages of CKD including individuals with CKD stage 1–2 (n = 37) and CKD stage 3–4 (n = 54), incident pre-dialysis CKD stage 5 patients (CKD5-ND; n = 386) and prevalent PD (n = 74) and HD (n = 195) patients. The demographic, clinical and biochemical characteristics of the study groups are presented in **Table 1**. The groups differed in regards to age with the youngest patients in the CKD5-ND group (median, 55 years) and the oldest in the CKD1-2 group (median, 68 years). Diabetes was most common in CKD stage 3–4 group (44%), and malnutrition (45%) and CVD (61%) most common among the HD patients. The highest level of inflammatory and oxidative stress biomarkers was also among HD patients (hsCRP: median 6.5 mg/L and 8-OHdG: median 1.3 ng/ml).

**Table 1:** Baseline characteristics and biochemical parameters in all individuals participating in the study.

	CKD 1–2 (n = 37)	CKD 3–4 (n = 54)	CKD5-ND (n = 386)	HD (n = 195)	PD (n = 74)
Age (year)^1^	68 (48–79)	60 (34–76)	55 (34–68)	64 (38–80)	61 (31–80)
Males (%)	70	74	60	57	64
DM (%)^1^	0	44	28	23	20
CVD (%)^1^	8	26	31	61	22
BMI (kg/m^2^)	25 (20–29)	25 (20–32)	24 (20–30)	24 (18–30)	25 (19–29)
SGA>1 (%)^1^	6	21	30	45	42
Albumin (g/L)^1^	39 (35–42)	37 (28–41)	34 (26–41)	35 (29–41)	32 (26–37)
eGFR (ml/min/1.73^2^); CKD-EPI^1^	79 (68–111)	27 (15–43)	6 (4–10)	0*	6 (4–9)
Creatinine (mg/dl)^1^	0.9 (0.7–1.1)	2.5 (1.6–3.8)	7.7 (5.1–11.6)	8.3 (5.7–11.4)	7.7 (5.3–11.1)
Cholesterol (mmol/L)^1^	5.0 (4.2–6.1)	5.2 (3.8–6.9)	5.1 (3.4–7.3)	4.3 (3.1–5.8)	4.9 (3.7–6.7)
hsCRP (mg/L)^1^	1.2 (0.4–4.2)	3.1 (0.5–14)	4.2 (0.6–30.6)	6.5 (0.6–46.0)	2.7 (0.3–21.4)
IL-6 (pg/ml)^1^	1.9 (0.5–5.9)	3.3 (1.5–9.9)	6.1 (1.6–16.8)	8.1 (2.6–30.4)	4.9 (0.9–18.3)
Statins (%)^1^	0	30	21	32	35
sVCAM-1 (ng/ml)^1^	689 (500–949)	902 (705–1591)	1286 (825–1900)	1679 (1156–2852)	-
8-OHdG (ng/ml)^1^	0.3 (0.0–0.5)	0.5 (0.2–0.8)	0.7 (0.3–1.1)	1.3 (0.5–2.1)	0.5 (0.2–0.7)
Pentosidine (nmol/L)^1^	243 (178–319)	400 (247–882)	994 (469–2196)	1728 (616–3028)	673 (350–1763)
Pentosidine, nmol/g albumin^1^	6.5 (4.6–8.8)	11.1 (6.3–35.3)	30.7 (14.1–63.4)	48.4 (19.1–84.8)	22.7 (10.4–57.9)

Values are expressed as median (10–90 percentiles) or percentage. eGFR, glomerular filtration rate calculated by CKD-EPI equation; DM, diabetes mellitus; CVD, cardiovascular disease; BMI, body mass index; SGA, subjective global assessment; hsCRP, high sensitivity C-reactive protein; IL-6, interleukin 6; sVCAM-1, soluble vascular endothelial cell adhesion molecule; 8-OHdG, 8-hydroxy-2'-deoxyguanosine.

*Most of HD patients were anuric or had a minimal urine production.

^**1**^ Significant difference between the groups, p value <0.05

**Fig 1:**
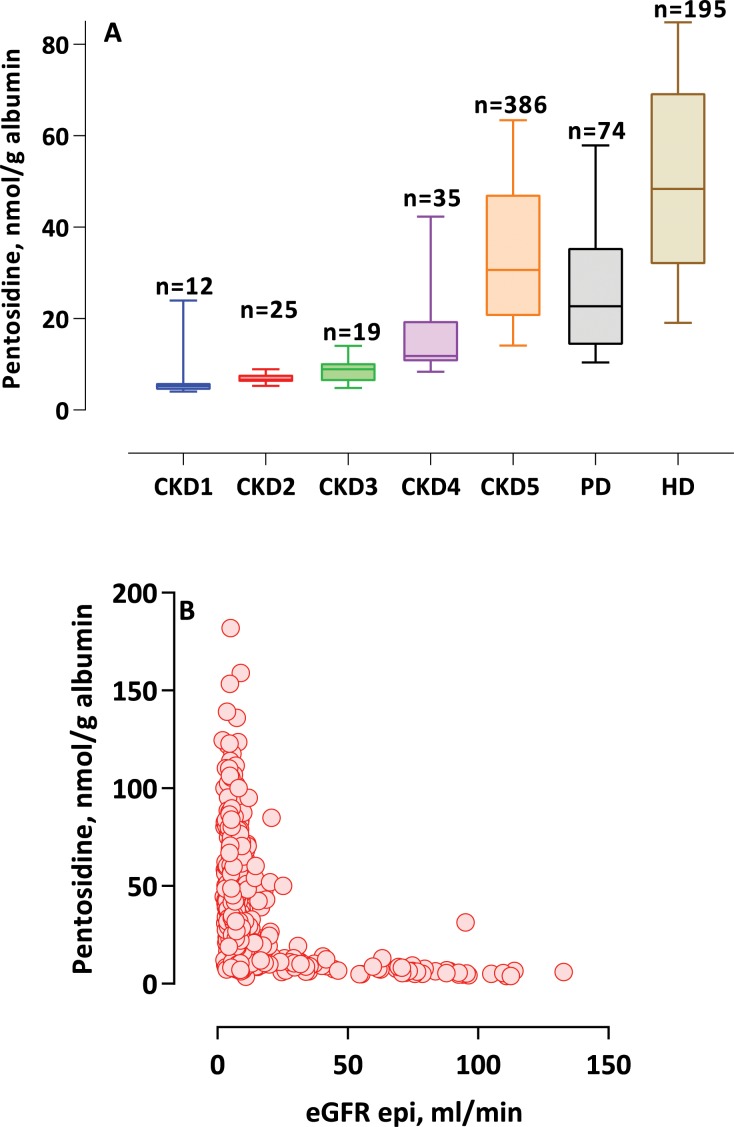
(A) Baseline plasma pentosidine levels, corrected for albumin level, for each studied cohort including all individuals (n = 746) and shown as box-and-whisker plots depicting median, and 10th to 90th percentile.(B)Correlation between plasma pentosidine level, corrected for albumin, and estimate glomerular filtration rate (eGFR) based on chronic kidney disease epidemiology collaboration (CKD-EPI) formula or in haemodialysis patients assuming eGFR zero in all investigated individuals (n = 746).

Pentosidine levels in each CKD stage 1–4, CKD5-ND and PD and HD patients are shown in **Fig 1A**. We could observe a trend of increasing pentosidine level with the progression of CKD stages. Compared to CKD stage 1 group, CKD5-ND, and PD and HD patients had significantly higher pentosidine levels. The level of pentosidine in PD patients was significantly lower compared to HD patients, but not different compared to CKD5-ND patients.

Subgroup analysis showed no significant difference in plasma pentosidine levels between DM and non-DM patients in any of the group or for the combined cohort (data not shown). In CKD stage 3–4 patients with DM, HbA1c was significantly associated with pentosidine (rho = 0.60, p<0.01) and also in non-DM patients (rho = 0.52, p<0.01), while in CKD 1–2 there was no DM patients and in HD patients there were no available measurements of HbA1c. Age associated with pentosidine concentrations which for the age groups ≤45 years, 45–65 years and >65 years were respectively 24.5 (10.6–56.3), 31.9 (10.4–75.2) and 37.6 (8.5–83.0) nmol/g albumin (p<0.0001). The pentosidine also associated with the aetiology of CKD and was highest in patients with hypertension/renal vascular disease, 40.6 (18.3–84.3) nmol/g albumin which was higher than in GN patients, 26.9 (9.2–64.4) nmol/g albumin (p<0.01), non-significantly different compared to DM, 33.0 (14.2–71.2) nmol/g albumin (p = 0.07) and higher compared to other aetiologies, 31.8 (11.3–76.0) nmol/g albumin (p<0.01). The correlation between pentosidine (both corrected for albumin and not corrected for albumin) and albuminuria in 250 patients in whom albuminuria was analyzed: 38 patients with CKD 3–4 and 212 CKD 5-ND patients had albuminuria of 1542.5 (145.3–6094.6) and 352.5 (4.0–2001.7) mg/L per 24h respectively. There was no significant correlation between pentosidine and albuminuria (rho = 0.03, p = 0.60) while there was a significant correlation between pentosidine corrected for albumin and albuminuria (rho = 0.14, p = 0.03).

### Medications and plasma pentosidine levels

Overall 25% of the patients received treatment with statins. In CKD 1–2 group, none of the individuals was taking statins. In the other groups there were no significant differences in pentosidine levels in patients who had statins compared to those did not receive statins. Also there was no significant difference (p = 0.57) in pentosidine levels between patients who were on ACEI or ARB treatment and those who were not: median 33.0 (11.8–71.6) versus without 32.1 (11.9–76.5) nmol/g albumin. Iron therapy was given to 62% of the patients with major differences between the groups: CKD 1–2 (0%), CKD 3–4 (54%), CKD5-ND (65%), HD (67%) and PD (29%). When analysing the possible impact of iron on pentosidine in HD patients who had the highest exposure to iron, plasma pentosidine levels did not differ significantly (p = 0.87) between those who received versus those who did not receive iron therapy: median 53.8 (18.5–85.6) versus median 50.2 (25.0–85.0) nmol/g albumin.

### Univariate correlations of pentosidine with other biomarkers

In individuals with CKD 1–2, pentosidine correlated with age (rho = 0.48, p<0.01), sVCAM-1 (rho = 0.34, p<0.05) and, inversely, with GFR (rho = -0.58, p<0.001). In CKD stage 3–4 patients, pentosidine correlated with sVCAM-1 (rho = 0.33, p<0.05), 8-OHdG (rho = 0.38, p<0.05) and, inversely, with GFR (rho = -0.60, p<0.001). Among CKD5-ND patients, pentosidine correlated with age (rho = 0.21, p<0.001), 8-OHdG (rho = 0.37, p<0.001), hsCRP (rho = 0.21, p<0.001), IL-6 (rho = 0.24, p<0.001) and sVCAM-1 (rho = 0.30, p<0.001), and inversely with GFR (rho = -0.24, p<0.001). In PD patients, pentosidine correlated with 8-OHdG (rho = 0.37, p<0.001), Il-6 (rho = 0.27, p<0.05) and negatively with GFR (rho = -0.35, p<0.01). Finally, in HD patients, pentosidine was correlated with age (rho = 0.27, p<0.01), IL-6 (rho = 0.15, p<0.05) and BMI (rho = -0.16, p<0.05). Note that whereas most HD patients had minimal RRF or were anuric, we lack quantitative data on RRF or GFR in the prevalent HD patients.

The relation of pentosidine with GFR was further assessed in all 746 investigated individuals, based on calculation of estimated GFR (eGFR) using the CKD-EPI equation and assuming a value of zero for the prevalent HD patients (**Fig 1B**).

In the combined cohort of all investigated individual (n = 746) there were significant (p<0.0001) correlations between plasma pentosidine and markers of inflammation (hsCRP, IL-6), oxidative stress (8-OHdG) and eGFR.

### Multivariate analysis of determinants of plasma pentosidine

Results of multivariate linear regression analysis of factors associated with 1-SD higher pentosidine in all 746 individuals are shown in **Table 2**. Higher age, malnutrition (SGA), oxidative stress (8-OHdG, 1-SD higher), and belonging to the CKD5-ND, HD and PD cohorts had a significant association with pentosidine after adjusting for sex, presence of DM, and inflammation (hsCRP). The applied model (**Table 2**) shows to what extent having ESRD (CKD5-ND, HD and PD cohorts) vs being in CKD 1–2 stage associates with 1-SD higher pentosidine level and demonstrates that being an ESRD patient explains much of the variation in pentosidine level in parallel with higher age, malnutrition and oxidative stress.

**Table 2:** Predictors of one standard deviation (1-SD) higher plasma pentosidine concentration according to the imputed multivariate linear regression analysis in 746 patients.

Total (n = 746, adjusted r^2^ = 0.25)	Beta	T values	P value
**Age, years (1-SD)**	**0.13**	**3.82**	**<0.0001**
Gender, male versus female	0.02	0.62	0.54
DM, presence versus absence	-0.03	-1.0	0.31
**SGA, malnourished versus well nourished**	**0.07**	**2.32**	**0.02**
hsCRP, mg/L (1-SD)	0.03	0.87	0.38
**8-OHdG, ng/ml (1-SD)**	**0.13**	**3.06**	**<0.01**
CKD 3–4 patients versus CKD 1–2	0.07	1.51	0.13
**CKD5-ND patients versus CKD 1–2**	**0.56**	**7.02**	**<0.0001**
**HD patients versus CKD 1–2**	**0.62**	**8.07**	**<0.0001**
**PD patients versus CKD 1–2**	**0.23**	**4.40**	**<0.0001**

DM, diabetes mellitus; SGA, subjective global assessment of nutritional status; hsCRP, high-sensitivity C-reactive protein; 8-OHdG, 8-hydroxy-2'-deoxyguanosine.

### Pentosidine level, all-cause and CVD mortality

The relative risk of death, occurring within 60 months, in the combined group of all individuals (n = 746) was significantly associated with 1-SD higher pentosidine (RR = 1.04; 95% CI, 1.01–1.08, p = 0.01) after adjustment for confounders, i.e., age, gender, CVD, DM, SGA, hsCRP (1-SD higher), 8-OHdG (1-SD higher) and cohort (**Table 3**). In addition, relative risk of death due to CVD events was significantly associated with 1-SD higher pentosidine (RR = 1.03; 95% CI, 1.01–1.06, p = 0.03) after adjustment for the same confounders (**Table 4**).

**Table 3:** The all-cause mortality risk for death occurring within 60 months based on imputed data in the combined cohort of 746 individuals, adjusted for all confounders, and expressed as relative risk ratio (95% confidence interval, CI).

Variable	Relative risk (95% CI)	P value
**Pentosidine, nmol/L (1-SD)**	**1.04 (1.01–1.08)**	**0.01**
**Age, years (1-SD)**	**1.08 (1.04–1.11)**	**<0.0001**
Gender, male versus female	1.04 (0.98–1.10)	0.25
**CVD, presence versus absence**	**1.15 (1.07–1.24)**	**0.0001**
**DM, presence versus absence**	**1.13 (1.05–1.21)**	**<0.001**
**SGA, malnourished versus well nourished**	**1.16 (1.09–1.24)**	**<0.0001**
hsCRP, mg/L (1-SD)	1.02 (0.99–1.06)	0.18
**8-OHdG, ng/ml (1-SD)**	**1.04 (1.01–1.09)**	**0.03**
CKD 3–4 versus CKD 1–2	1.09 (0.92–1.30)	0.33
CKD5-ND versus CKD 1–2	1.08 (0.93–1.25)	0.34
HD patients versus CKD 1–2	1.09 (0.92–1.29)	0.34
PD patients versus CKD 1–2	1.14 (0.97–1.35)	0.12

CVD, cardiovascular disease; DM, diabetic mellitus; SGA, subjective global assessment of nutritional status; hsCRP, high-sensitivity C-reactive protein; 8-OHdG, 8-hydroxy-2'-deoxyguanosine.

**Table 4:** The cardiovascular mortality risk for death occurring within 60 months based on imputed data in the combined cohort of 746 individuals, adjusted for all confounders, and expressed as relative risk ratio (95% confidence interval, CI).

Variable	Relative risk (95% CI)	P value
**Pentosidine, nmol/L (1-SD)**	**1.03 (1.01–1.06)**	**0.03**
Age, years (1-SD)	1.01 (0.98–1.05)	0.22
Gender, *male versus female*	0.99 (0.94–1.04)	0.75
**CVD, presence versus absence**	**1.19 (1.12–1.26)**	**0.0001**
**DM, presence versus absence**	**1.12 (1.06–1.19)**	**<0.0001**
**SGA, malnourished versus well nourished**	**1.09 (1.03–1.15)**	**0.002**
hsCRP, mg/L (1-SD)	1.01 (0.98–1.04)	0.38
8-OHdG, ng/ml (1-SD)	1.03 (0.99–1.06)	0.06
CKD 3–4 versus CKD 1–2	1.05 (0.91–1.22)	0.44
CKD5-ND versus CKD 1–2	1.08 (0.93–1.25)	0.93
HD patients versus CKD 1–2	0.95 (0.83–1.10)	0.52
PD patients versus CKD 1–2	1.04 (0.91–1.20)	0.51

CVD, cardiovascular disease; DM, diabetic mellitus; SGA, subjective global assessment of nutritional status; hsCRP, high-sensitivity C-reactive protein; 8-OHdG, 8-hydroxy-2'-deoxyguanosine.

A separate analysis in non-dialyzed (n = 477) and dialyzed (n = 269) patients respectively (see **S1–S4 Tables**) using the same method and variables showed that in non-dialyzed patients pentosidine was not an independent predictor of all-cause mortality, RR = 1.02 (CI, 0.98–1.05, p = 0.44), but an independent predictor of CVD mortality, RR = 1.03 (CI, 1.01–1.07, p = 0.03). In contrast, in the dialyzed population (HD plus PD), pentosidine was an independent predictor of all-cause mortality, RR = 1.09 (CI, 1.02–1.15, p<0.01), but not of CVD-mortality, RR = 1.02 (CI, 0.97–1.08, p = 0.41), see **S1–S4 Tables**.

**Fig 2:**
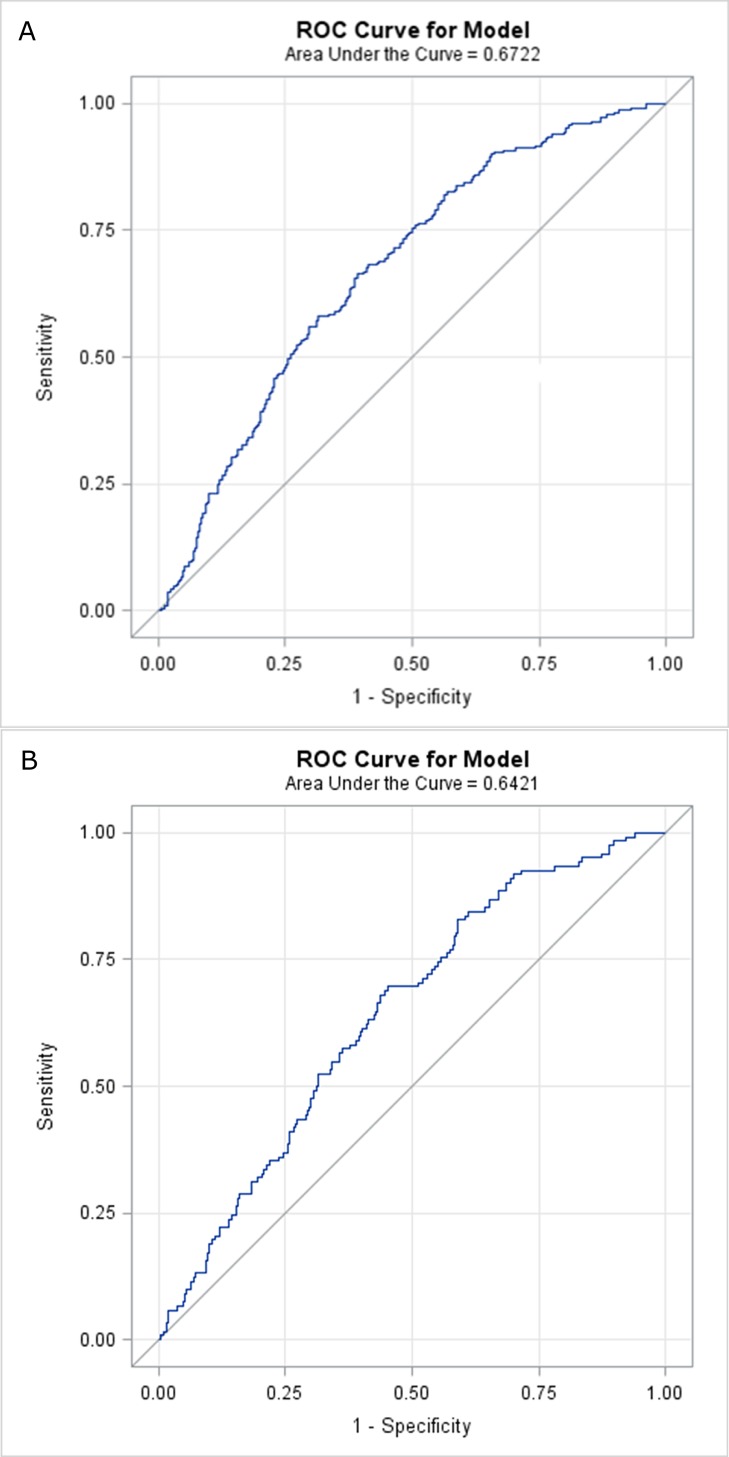
(A) Areas under the curves (AUC) of receiver operating characteristics (ROC) in 746 patients for pentosidine in relation to all-cause mortality.(B) Areas under the curves (AUC) of receiver operating characteristics (ROC) in 746 patients for pentosidine in relation to CVD-mortality.

To establish the sensitivity and specificity of pentosidine as a prognostic biomarker, ROC curves for pentosidine versus all-cause mortality and CVD mortality were investigated. The cut-off point for pentosidine in predicting all-cause mortality was 34.6 nmol/g albumin whereas for CVD-mortality it was 37.6 nmol/g albumin. The ROC curves are presented in **Fig 2** above and **2 below**.

## Discussion

Plasma pentosidine levels which were several folds higher among ESRD patients (CKD5-ND, PD and HD patients) as compared to individuals with CKD stage 1, 2, 3 and 4, associated with low GFR and increased levels of biomarkers of oxidative stress and inflammation. Interestingly, a difference appeared to be present between dialysis modalities: HD patients had significantly higher pentosidine levels compared to PD patients (see **Fig 1A**). The latter had higher RRF which associates with lower circulating pentosidine (see **Fig 1B**); however, it cannot be excluded that dialysis modality could also play a role for the observed difference in pentosidine levels between HD and PD patients.

A higher plasma pentosidine concentration associated with lower GFR within each of the separate cohorts, and in the combined cohort comprising 746 patients (**Fig 1B**). In addition, age, malnutrition, oxidative stress (8-OHdG) and group entities were significant predictors of a 1-SD higher pentosidine level. Thus, whereas variations in plasma pentosidine concentrations may to a large extent be explained by RRF, also higher age, an increased pro-oxidative state, and malnutrition, which are typical findings in uremic patients, as well as characteristics specific for each CKD stage or dialysis modality may also play a role.

The indicator of increased oxidative stress (8-OHdG), was positively correlated with pentosidine in most of the groups (PD, CKD stage 3–4 and CKD5-ND) and associations with biomarkers of inflammation was seen among CKD5-ND (IL-6 and hsCRP), HD (IL-6) and PD (IL-6) patients.

One of the factors that potentially may influence plasma pentosidine is usage of statins. A large number of randomized control trials have shown that statins help in the primary and secondary prevention of cardiovascular events, not only via their lipid-lowering effect, but also due to their anti-inflammatory potential [23]. In our study we did however not find a significant relationship between statins and pentosidine level. Similarly, the use of ACEI or ARB did not seem to influence pentosidine level.

There were no significant differences in plasma pentosidine between diabetics and non-diabetic patients in any of our studied cohorts and only in diabetic and non-diabetic patients with CKD 3–4 there was a significant correlation between plasma pentosidine and HbA1c. This may suggest that the state of hyperglycemia is influencing plasma pentosidine concentration only at the early stages of CKD, while with further progression of CKD, factors associated with renal failure and uremia are more important contributors to increased plasma pentosidine than hyperglycemia.

Furthermore, in multivariate analysis, diabetic status was not a significant predictor of the pentosidine level (**Table 2**). This again may suggest that enhanced production and accumulation of AGEs among patients with impaired renal function is mainly related to conditions other than hyperglycaemia [24], possibly because the effect of glycation is diminished by the effect of kidney failure and uremia [7].

Thus, levels of AGEs in circulation, and in tissues such as skin, in patients with more advanced CKD apart from hyperglycaemia are influenced by factors such as oxidative stress, inflammation and decreased kidney clearance of AGE precursors [25] which may explain why AGEs like pentosidine increase [26] and concentrations of pentosidine and creatinine correlate with a decline of renal function [7, 26]. In PD patients, plasma pentosidine was shown to negatively correlate with RRF, emphasizing the importance of maintaining renal function in reducing carbonyl and oxidative stress [8].

Noteworthy, circulating AGEs may not accurately reflect the total body burden of AGEs; instead measurements of tissue AGEs could be more relevant. As a consequence of the increased peritoneal glucose exposure from PD fluids, the content of AGEs in the peritoneum was reported to be increased in PD patients [27] suggesting that tissue AGEs do not necessarily depend only on circulating pentosidine. Recently, tissue AGEs of the skin can be measured by skin autofluorescence (SAF) with a device that uses the fluorescent properties of AGEs to estimate the level of AGE accumulation in the skin [28]. Using such a device, we found no significant association between SAF and plasma pentosidine in the PD group (data not shown).

The survival analysis showed that 1-SD higher pentosidine was a significant, independent predictor of all-cause and CVD mortality after adjustment for confounders. Circulating pentosidine associates with progression of atherosclerosis indicated by changes in carotid intima-media thickness during the first year of PD and HD therapy [9]. HD patients with higher plasma pentosidine had increased risk for cardiovascular events [10], accelerated rate of progression of aortic stiffness [11] and exhibited negative association with carotid distensibility suggesting its role in the development of arterial stiffness [12]. However, other studies showed no relationship between plasma pentosidine, intima media thickness and the number of atherosclerotic plaques [13]. The contribution of pentosidine to the development of cardiovascular events and mortality in CKD patients is still disputed [14]. Several prospective studies have reported positive association between plasma AGEs and mortality in populations with or without diabetes type 2 [29–31]. A study among patients with type-1 diabetes showed that high plasma AGEs including pentosidine associated with fatal and non-fatal CVD as well as with increased all-cause mortality [32]. However, a study conducted among HD patients, did not find that high levels of CML and total serum fluorescence indicating AGEs were associated with all-cause and cardiovascular mortality [33]. We have previously reported that in patients starting on dialysis circulating pentosidine correlated with biomarkers of inflammation and malnutrition, but not with all-cause mortality [6], while, in HD patients, IL-6 was found to be a better predictor of mortality than pentosidine and other oxidative stress and inflammatory biomarkers [34]. However the current study comprising a larger number of CKD patients establishes that plasma pentosidine indeed associates with increased mortality. On the other hand, the mortality predictive pattern seemed to differ between non-dialyzed patients in whom pentosidine was not an independent predictor of all-cause mortality but an independent predictor of CVD mortality, whereas in the dialyzed patients (HD plus PD), pentosidine was not an independent predictor of all-cause mortality but an independent predictor of CVD mortality. These intriguing results may suggest that in non-dialyzed CKD patients who have an increased risk of CVD mortality related to GFR; pentosidine may promote cardiovascular deaths whereas in ESRD patients undergoing dialysis with no or less variable renal function, other factors than pentosidine such as inflammation, malnutrition and fluid overload are more important as CVD risk factors.

This study has several limitations. Due to its cross-sectional design we cannot conclude on causality. Also, the measurement of pentosidine was done only at a single time point; therefore, we have no knowledge about variations of pentosidine level over time. In addition, this is a post hoc analysis which may decrease the generalizability of observations due to biases. On the other hand, to the best of our knowledge, this is the first study investigating determinants of plasma pentosidine—measured by an accurate HPLC method and corrected for albumin—across a wide spectrum of different CKD stages and dialysis modalities, and its association with cardiovascular and all-cause mortality.

In summary, plasma pentosidine is markedly elevated in patients with CKD in whom a high pentosidine concentration associated with low GFR, and signs of oxidative stress and inflammation. Perhaps contrary to expectations, considering the peritoneal glucose load which is thought to promote formation of AGEs, plasma pentosidine was lower in prevalent PD patients than in prevalent HD patients. Although this difference appears to be related mainly to the higher RRF among the PD patients, less oxidative stress and less inflammation in PD patients may also have played a role. High plasma pentosidine was found to be an independent predictor of all-cause and CVD mortality suggesting that it may represent a useful biomarker and a potential target for interventions aiming at improving clinical outcome in ESRD patients.

## Supporting Information

S1 Table The all-cause mortality risk for death occurring within 60 months based on imputed data in the combined cohort of 477 non-dialyzed patients, adjusted for all confounders, and expressed as relative risk ratio (95% confidence interval, CI). (DOCX)

S2 Table The cardiovascular mortality risk for death occurring within 60 months based on imputed data in the combined cohort of 477 non-dialyzed patients, adjusted for all confounders, and expressed as relative risk ratio (95% confidence interval, CI). (DOCX)

S3 Table The all-cause mortality risk for death occurring within 60 months based on imputed data in the combined cohort of 269 dialyzed patients, adjusted for all confounders, and expressed as relative risk ratio (95% confidence interval, CI). (DOCX)

S4 Table The cardiovascular mortality risk for death occurring within 60 months based on imputed data in the combined cohort of 269 dialyzed patients, adjusted for all confounders, and expressed as relative risk ratio (95% confidence interval, CI). (DOCX)
