# Supplementary material for: Plasma Pentosidine and Its Association with Mortality in Patients with Chronic Kidney Disease
Source: PLoS One. 2016 Oct 4;11(10):e0163826. doi: 10.1371/journal.pone.0163826 (PMC5049770; doi:10.1371/journal.pone.0163826)
Supplement: S1 Table — (DOCX) [file pone.0163826.s001.docx]

**S1 Table.** The all-cause mortality risk for death occurring within 60 months based on imputed data in the combined cohort of 477 non-dialyzed patients, adjusted for all confounders, and expressed as relative risk ratio (95% confidence interval, CI).

| **Variable** | **Relative risk (95% CI)** | **P value** |
| --- | --- | --- |
| **Pentosidine, nmol/L (1-SD)** | 1.02 (0.98 – 1.05) | 0.44 |
| **Age, years (1-SD)** | **1.10 (1.06 – 1.14)** | **<0.0001** |
| Gender, male versus female | 1.00 (0.93 – 1.07) | 0.99 |
| **CVD, presence versus absence** | **1.16 (1.07 – 1.26)** | **<0.001** |
| **DM, presence versus absence** | **1.19 (1.11 – 1.28)** | **<0.0001** |
| **SGA , malnourished versus well nourished** | **1.26 (1.17 – 1.37)** | **<0.0001** |
| hsCRP, mg/L (1-SD) | 1.02 (0.99 – 1.06) | 0.24 |
| 8-OHdG, ng/ml (1-SD) | 1.03 (0.99 – 1.07) | 0.13 |
| CKD 3-4 versus CKD 1-2 | 1.07 (0.92 – 1.26) | 0.38 |
| CKD5-ND versus CKD 1-2 | 1.09 (0.94 – 1.25) | 0.25 |

CVD, cardiovascular disease; DM, diabetic mellitus; SGA, subjective global assessment of nutritional status; hsCRP, high-sensitivity C-reactive protein; 8-OHdG, [8-hydroxy-2'-deoxyguanosine](http://www.ncbi.nlm.nih.gov/pubmed/19412858).
