# Supplementary material for: Plasma Pentosidine and Its Association with Mortality in Patients with Chronic Kidney Disease
Source: PLoS One. 2016 Oct 4;11(10):e0163826. doi: 10.1371/journal.pone.0163826 (PMC5049770; doi:10.1371/journal.pone.0163826)
Supplement: S2 Table — (DOCX) [file pone.0163826.s002.docx]

**S2 Table.** The cardiovascular mortality risk for death occurring within 60 months based on imputed data in the combined cohort of 477 non-dialyzed patients, adjusted for all confounders, and expressed as relative risk ratio (95% confidence interval, CI).

| **Variable** | **Relative risk (95% CI)** | **P value** |
| --- | --- | --- |
| **Pentosidine, nmol/L (1-SD)** | **1.03 (1.01 – 1.07)** | **0.03** |
| Age, years (1-SD) | 1.03 (0.99 – 1.06) | 0.08 |
| Gender, male versus female | 0.99 (0.94 – 1.05) | 0.89 |
| **CVD, presence versus absence** | **1.23 (1.15 – 1.32)** | **<0.0001** |
| **DM, presence versus absence** | **1.16 (1.09 – 1.23)** | **<0.0001** |
| **SGA , malnourished versus well nourished** | **1.13 (1.06 – 1.21)** | **<0.001** |
| **hsCRP, mg/L (1-SD)** | **1.04 (1.01 – 1.07)** | **0.02** |
| 8-OHdG, ng/ml (1-SD) | 0.99 (0.97 – 1.03) | 0.98 |
| CKD 3-4 versus CKD 1-2 | 1.04 (0.91 – 1.19) | 0.54 |
| CKD5-ND versus CKD 1-2 | 0.99 (0.89 – 1.12) | 0.94 |

CVD, cardiovascular disease; DM, diabetic mellitus; SGA, subjective global assessment of nutritional status; hsCRP, high-sensitivity C-reactive protein; 8-OHdG, [8-hydroxy-2'-deoxyguanosine](http://www.ncbi.nlm.nih.gov/pubmed/19412858).
