# Supplementary material for: Plasma Pentosidine and Its Association with Mortality in Patients with Chronic Kidney Disease
Source: PLoS One. 2016 Oct 4;11(10):e0163826. doi: 10.1371/journal.pone.0163826 (PMC5049770; doi:10.1371/journal.pone.0163826)
Supplement: S4 Table — (DOCX) [file pone.0163826.s004.docx]

**S4 Table.** The cardiovascular mortality risk for death occurring within 60 months based on imputed data in the combined cohort of 269 dialyzed patients, adjusted for all confounders, and expressed as relative risk ratio (95% confidence interval, CI).

| **Variable** | **Relative risk (95% CI)** | **P value** |
| --- | --- | --- |
| Pentosidine, nmol/L (1-SD) | 1.02 (0.97 – 1.08) | 0.41 |
| Age, years (1-SD) | 1.01 (0.96 – 1.06) | 0.68 |
| Gender, male versus female | 0.98 (0.90 – 1.07) | 0.67 |
| CVD, presence versus absence | 1.10 (0.99 – 1.22) | 0.06 |
| DM, presence versus absence | 1.06 (0.95 – 1.18) | 0.33 |
| SGA , malnourished versus well nourished | 1.02 (0.93 – 1.12) | 0.61 |
| hsCRP, mg/L (1-SD) | 0.98 (0.93 – 1.02) | 0.29 |
| 8-OHdG, ng/ml (1-SD) | 1.04 (0.98 – 1.10) | 0.22 |
| HD versus CKD 1-2 | 1.08 (0.90 – 1.29) | 0.42 |
| PD versus CKD 1-2 | 1.12 (0.95 – 1.31) | 0.17 |

CVD, cardiovascular disease; DM, diabetic mellitus; SGA, subjective global assessment of nutritional status; hsCRP, high-sensitivity C-reactive protein; 8-OHdG, [8-hydroxy-2'-deoxyguanosine](http://www.ncbi.nlm.nih.gov/pubmed/19412858).
